# *In vivo* Imaging of Mitochondrial Transport in Single-Axon Regeneration of Zebrafish Mauthner Cells

**DOI:** 10.3389/fncel.2017.00004

**Published:** 2017-01-24

**Authors:** Yang Xu, Min Chen, Bingbing Hu, Rongchen Huang, Bing Hu

**Affiliations:** Chinese Academy of Sciences Key Laboratory of Brain Function and Disease, and School of Life Sciences, University of Science and Technology of ChinaHefei, China

**Keywords:** mitochondrial transport, axon regeneration, *in vivo* imaging, Mauthner cells, laser axotomy, single cell

## Abstract

Mitochondrial transport is essential for neuronal function, but the evidence of connections between mitochondrial transport and axon regeneration in the central nervous system (CNS) of living vertebrates remains limited. Here, we developed a novel model to explore mitochondrial transport in a single Mauthner axon (M axon) of zebrafish with non-invasive *in vivo* imaging. To confirm the feasibility of using this model, we treated labeled zebrafish with nocodazole and demonstrated that it could disrupt mitochondrial transport. We also used two-photon laser axotomy to precisely axotomize M axons and simultaneously recorded their regeneration and the process of mitochondrial transport in living zebrafish larvae. The findings showed that the injured axons with stronger regenerative capability maintain greater mitochondrial motility. Furthermore, to stimulate axon regeneration, treatment with dibutyryl cyclic adenosine monophosphate (db-cAMP) could also augment mitochondrial motility. Taken together, our results provide new evidence that mitochondrial motility is positively correlated with axon regeneration in the living vertebrate CNS. This promising model will be useful for further studies on the interaction between axon regeneration and mitochondrial dynamics, using various genetic and pharmacological techniques.

## Introduction

Mitochondria are dynamic organelles (Misgeld et al., 2007; Saxton and Hollenbeck, 2012; Schwarz, 2013; Sheng, 2014) that play an essential role under different physiological conditions in neurons, such as synaptic plasticity (Li et al., 2004), axon degeneration (Court and Coleman, 2012; O’Donnell et al., 2013), and growth cone guidance (Steketee et al., 2012; Lathrop and Steketee, 2013). Considering the complex structure and morphology of neurons, appropriate distribution and movement of mitochondria in somata, axons, and dendrites are vital for neuronal maintenance and function. Changes in mitochondrial dynamics have been implicated in regeneration following axonal injury (Misgeld et al., 2007; Lathrop and Steketee, 2013; Zhou et al., 2016). Numerous studies have paid much attention to *in vivo* imaging in vertebrate organisms of single-axon regeneration (Kerschensteiner et al., 2005; Canty et al., 2013; Lorenzana et al., 2015) and mitochondrial transport in axons (Misgeld et al., 2007; Plucinska et al., 2012; O’Donnell et al., 2013; Sorbara et al., 2014). However, little is known about the relationship between single-axon regeneration and internal mitochondrial transport; specifically, there is a lack of non-invasive *in vivo* evidence in the vertebrate central nervous system (CNS).

Two prerequisites need to be satisfied to solve this problem. The first is to find a suitable vertebrate model that can monitor mitochondrial transport in the CNS at a single-cell level by non-invasive *in vivo* imaging. Researchers have presented several models to visualize mitochondrial transport *in vivo*. Taking invertebrate models (Pilling et al., 2006; Russo et al., 2009) as an example, their nervous systems are mostly distinct from their counterparts in vertebrate organisms. With regard to vertebrate models, recent studies have begun to use mouse and zebrafish for intravital imaging of mitochondrial trafficking. However, *in vivo* imaging of mice requires invasive approaches because surgery is necessary before imaging (Misgeld et al., 2007; Sajic et al., 2013; Sorbara et al., 2014; Zhou et al., 2016). When it comes to zebrafish, previous studies were mainly focused on the peripheral nervous system (PNS) (Plucinska et al., 2012; O’Donnell et al., 2013) or particular types of neurons in CNS transgenic lines they labeled (Bergamin et al., 2016; Dukes et al., 2016). Despite these advances, non-invasive *in vivo* imaging of axonal transport of mitochondria in vertebrate CNS at a single-cell level remains limited. The second factor is that the vertebrate model should have regenerative ability. However, it is commonly acknowledged that in the majority of mammals, CNS regeneration is extremely difficult after axonal injury (Chen and Zheng, 2014). Hence, non-invasive *in vivo* observation of axon regeneration in the vertebrate CNS at the single-axon levels remains to be established.

Considering the zebrafish larvae as an optically accessible model, it is definitely suitable for monitoring mitochondrial transport in the CNS via non-invasive *in vivo* imaging. On the other hand, the CNS axons of zebrafish can generally regenerate after injury. To address above prerequisites, we developed a novel model to directly study mitochondrial dynamics in zebrafish Mauthner cells (M cells) through non-invasive *in vivo* imaging. M cells are a pair of easily identifiable, myelinated spinal cord neurons with large somata and long axons through the spinal cord (Korn and Faber, 2005). Following laser axotomy, we first found that M cell axons have strong regenerative capability. This characteristic provided us unique opportunities to observe spinal nerve regeneration after axonal injury. Using single-cell electroporation and a Gal4- upstream activating sequence (UAS) system (Haas et al., 2001; Bestman et al., 2006; Distel et al., 2009; Plucinska et al., 2012; Huang et al., 2015), we could specifically label axonal mitochondria in M cells, focusing on their mitochondrial transport in the process of axon regeneration. We demonstrated close connections between axon regeneration and mitochondrial motility in the M cells of zebrafish larvae. Meanwhile, pharmacological manipulations revealed that dibutyryl cyclic adenosine monophosphate (db-cAMP) (N6,2′-*O*-dibutyryladenosine 3′:5′-cyclic monophosphate sodium salt) enhanced axon regeneration and improved mitochondrial motility. Collectively, our findings support a promising model for exploring mitochondrial transport in living vertebrate CNS systems by non-invasive *in vivo* imaging and illustrate that mitochondria may play a crucial role in the process of axon regeneration.

## Materials and Methods

### Animal Care

Zebrafish (*Danio rerio*) wild-type *AB* lines and transgenic *Tg (Tol-056)* lines (Satou et al., 2009) were maintained on a 14/10-h light-dark cycle at 28.5°C. To avoid pigment formation, embryos were maintained in embryo medium containing 0.2 mM *N*-phenylthiourea (PTU, Sigma) after 24 h post-fertilization (hpf). Until 4 days post-fertilization (dpf), zebrafish larvae were reared on living paramecia. Both males and females were randomly used. All animal experiments were in accordance with the guidelines and regulations approved by the University of Science and Technology of China (USTC) Animal Resources Center and University Animal Care and Use Committee. The protocol was approved by the Committee on the Ethics of Animal Experiments of the USTC (Permit Number: USTCACUC1103013).

### Single-Cell Electroporation

To simultaneously label M cells and their mitochondria in living zebrafish larvae, plasmids PCS2^+^-CMV-Gal4-vp16, UAS-mito-enhanced green fluorescent protein (UAS-mito-EGFP), and UAS-DsRed2 were co-transfected through electroporation from DNA-filled micropipettes. Briefly, we cloned Gal4-vp16 (a gift from Jiulin Du, Shanghai Institutes for Biological Sciences, Shanghai, China) into the PCS2^+^ vector by cutting with BamHI and XhoI and used the CMV promoter to drive Gal4-vp16 expression. The full mito-EGFP sequence was a fragment that encodes a mitochondrial targeting sequence from subunit VIII of human cytochrome c oxidase (Misgeld et al., 2007; Kim et al., 2008) and EGFP. This mito-EGFP was cloned into a UAS expression vector (digest with EcoRI and NotI; a gift from Jiulin Du, Shanghai Institutes for Biological Sciences, Shanghai, China) with an E1b promoter to generate UAS-mito-EGFP. DsRed2 cloned from pDsRed2-C1 (Clontech) was added to a UAS expression vector to obtain UAS-DsRed2.

At 4 dpf, zebrafish AB/wildtype larvae were anesthetized using 0.02% tricaine methanesulfonate (MS222, Sigma) and embedded in 1% low-melting agarose for electroporation. Borosilicate micropipettes (resistance from 20 to 30 MΩ) were filled with mixed solution containing plasmids (PCS2^+^-CMV-Gal4-vp16, UAS-mito-EGFP and UAS-DsRed2; about 200 ng/μL each, dissolved in deionized water) and Alexa Fluor 488 hydrazide (Molecular Probes). When inserted into the zebrafish brain, the micropipette tip was pushed against the M soma. The mixture was electroporated into somata, applying 14–16 V pluses with durations of 0.5 ms at a frequency of 100 Hz; these were generated by an Axoporator 800A (Molecular Devices) (Bestman et al., 2006; Huang et al., 2015). At 48 h after electroporation (zebrafish larvae at 6 dpf), labeled M cells could be screened for fluorescent labeling with mito-EGFP and DsRed2.

### Nocodazole Treatment

Nocodazole is a typical inhibitor of microtubule polymerization that can effectively restrict mitochondrial transport (Plucinska et al., 2012). To prove that the model is useful for visualizing real mitochondrial transport *in vivo* in zebrafish M cells, larvae labeled with fluorescent compounds at 6 dpf were treated with nocodazole (Sigma) at a final concentration of 200 nM dissolved in embryo medium containing 0.2 mM PTU and 1% dimethyl sulfoxide (DMSO, Sigma) (Plucinska et al., 2012). The zebrafish larvae were maintained in the medium for 7 h before imaging. Control zebrafish larvae were maintained in embryo medium containing 0.2 mM PTU and 1% DMSO without nocodazole for 7 h before imaging.

### Two-Photon Axotomy

Zebrafish larvae were anesthetized in 0.02% MS222 at 6 dpf and mounted in 1% low-melting agarose prior to axotomy. Then the ablated site of M axons over cloacal pores (**Figure 3A**) were scanned and focused under a Zeiss microscope (LSM710, Germany) equipped with a 25X water-immersion objective (N.A. 0.8). Next, 800 nm two-photon laser at a laser intensity of 15% transmission was used to transect axons over ∼1.5 s (O’Brien et al., 2009; O’Donnell et al., 2013).

### Microinjection

It was previously shown that db-cAMP (Sigma) can stimulate injured M-axons to regenerate in living zebrafish (Bhatt et al., 2004), but it is not known whether it affects mitochondrial transport in regenerated axons. To evaluate the effects of db-cAMP on mitochondrial transport, micropipettes filled with 20 mM db-cAMP (dissolved in deionized water) or vehicle (deionized water) were microinjected (about 14 psi) with 0.5 nL into zebrafish hindbrain at 8 dpf. The micropipette tips were placed near the M soma, and pressure was applied with a Microinjection Dispense System (Picospritzer III, Parker, CO, USA). The zebrafish larvae were imaged within 10–30 min after microinjection. In axon regeneration experiments, zebrafish larvae were microinjected with 20 mM db-cAMP or vehicle every day from 6 to 7 dpf and imaged at 8 dpf. Microinjected zebrafish larvae survived and developed normally.

### *In vivo* Imaging of Axons and Mitochondria

All images were taken from lateral views of zebrafish, anterior to the left, and dorsal toward the top. For observing axon regrowth after two-photon axotomy, anesthetized zebrafish larvae were imaged at 1–4 days post-axotomy (dpa) (1–4 dpa is equivalent to 7–10 dpf of zebrafish larvae) using an Olympus FV1000 confocal microscope (Olympus, Japan) equipped with a 40× water-immersion objective (N.A. 0.8) at 2-μm steps.

To monitor mitochondrial transport, electroporated zebrafish larvae were imaged on different days for experimental requirements using a confocal microscope with a 60× water-immersion objective (N.A. 1.1). Mitochondrial transport in the db-cAMP experiment was imaged at 8 dpf. In axon regeneration experiments, mitochondrial transport was imaged from 1 to 4 dpa. Two axonal areas were selected for time-lapse imaging of each zebrafish: one was the area of proximal axons immediately adjacent to the M soma within 200 μm, and the other was the area of distal axons within 200 μm proximal to the site of axons over cloacal pores. For each area, 2.5-min movies were acquired with an imaging frequency about 1.5 s, and the imaging length of axons was ∼43 μm. After imaging, the zebrafish larvae were released from the low-melting agarose to recover.

### Image Processing and Analysis

To analyze the length of regenerated axons, 3D datasets were transformed into 2D projections of confocal stacks using FV10-ASW 4.2 viewer software (Olympus, Japan), and the images were assembled with Photoshop CS4 (Adobe, USA). The increased length of axons after injury was calibrated to convert pixels into distance using FV10-ASW 4.2 viewer software. Note that the starting point of calculation was the ablated site of axons over cloacal pores, and the axonal terminal of regeneration was defined as the end point of regrowth axons. The net length of regenerative axons on a measurement day refers to increased axonal lengths the day before the measurement day.

Processing and analysis to quantify mitochondrial transport were performed as previously described (Miller and Sheetz, 2004; Misgeld et al., 2007; Plucinska et al., 2012; O’Donnell et al., 2013; Bolea et al., 2014). All images were processed using Fiji/ImageJ software (National Institutes of Health). Mitochondrial motility is the percentage of moving mitochondria during a 2.5-min time-lapse movie in a given field of axonal segments (O’Donnell et al., 2013; Paquet et al., 2014). To measure features of moving mitochondria, *plugins/manual tracking* was used to track individual mitochondria only if they moved at least 2 μm (Misgeld et al., 2007; O’Donnell et al., 2013). Speed refers to the total moving distance of a mitochondrion divided by its observed moving time. Transport direction is the percentage of anterograde mitochondria, defined as the number of anterograde mitochondria divided by the total number of moving mitochondria.

### Statistical Analysis

The data were analyzed with GraphPad Prism 6 software (USA). Student’s *t*-tests and two-way analyses of variance (ANOVA) were used as indicated in the results. The results are presented as mean ± standard error of the mean (SEM), and *p*-values < 0.05 were considered significant.

## Results

### Monitoring Axonal Mitochondria of a Single M Cell in Living Zebrafish Larvae

**FIGURE 1:**
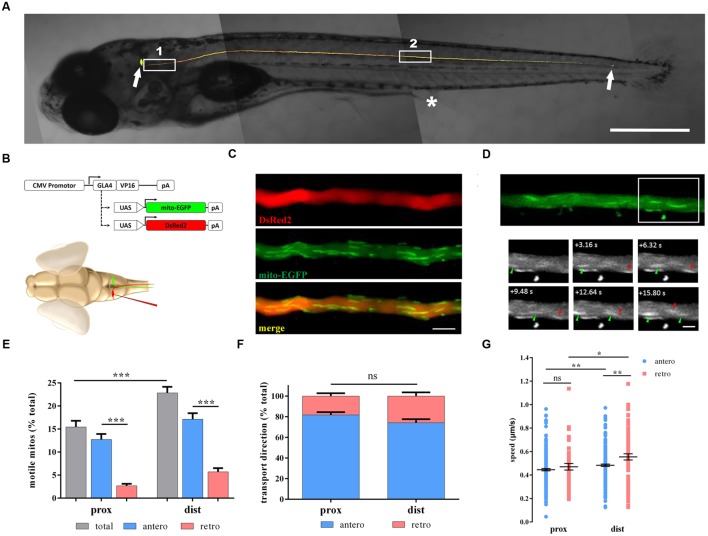
**Non-invasive *in vivo* imaging of mitochondrial transport in zebrafish M cells.** **(A)** M cells of zebrafish larvae were labeled with fluorescent proteins by single-cell electroporation at 6 days post-fertilization (dpf). White arrows indicate the M soma (leftward) and the M axon terminal (rightward). White box 1, proximal axon area; White box 2, distal axon area. Asterisk, cloacal pores. **(B)** Schematic of constructs used to label M cells (DsRed2) and mitochondria (mito-EGFP) through single-cell electroporation in living zebrafish larvae. **(C)** The labeled M axon was imaged with a confocal microscope, showing multiple tracts of mitochondria (mito-EGFP, middle) in the axon (DsRed2, top); merged image, bottom. **(D)** Moving mitochondria were monitored in the axon (area in white box). Arrowheads, moving mitochondria (anterograde, green; retrograde, red). **(E–G)** Mitochondrial motility **(E)**, transport direction (**F**; anterograde, blue; retrograde, pink), and speed **(G)** in axons at 7 dpf. Scale bars: **(A)** 500 μm, **(C)** 5 μm, **(D)** 2.5 μm. ^∗^*p* < 0.05, ^∗∗^*p* < 0.01, ^∗∗∗^*p* < 0.001. Error bars represent SEM.

We utilized multiple methods to genetically label mitochondria in M cells. The mitochondrial targeting sequence from cytochrome c oxidase was fused to the EGFP (mito-EGFP) to label axonal mitochondria (Kim et al., 2008; Plucinska et al., 2012) and cloned to UAS expression vectors. We could simultaneously label M cells and their mitochondria because of the co-expression of UAS-mito-EGFP and UAS-DsRed2 using the CMV promoter to drive the expression of Gal4-vp16. Gal4-UAS expression systems have already been proven to be suitable and non-toxic for zebrafish (Kim et al., 2008; Distel et al., 2009; Plucinska et al., 2012). In order to investigate the M axon and their mitochondria in living zebrafish larvae, we co-transfected these plasmids through single-cell electroporation into the zebrafish M soma (**Figure 1B**). The labeled single M cells could be detected 48 h later (**Figure 1A**), and the labeled mitochondria in the M axon of living zebrafish larvae could be non-invasively imaged with a confocal microscope (**Figures 1C,D**).

To identify whether axon position could affect mitochondrial transport, we chose two areas: proximal and distal axons (**Figure 1A**). Mitochondrial motility, transport direction, and speed were measured at 7 dpf in both areas. The motility of mitochondria in distal axons was apparently higher than that in proximal axons (prox: 15.5 ± 1.3% vs. dist: 22.8 ± 1.3%, *n* = 21 fish, Student’s two-tailed *t*-test, *p* < 0.001; **Figure 1E**). As for motile mitochondria, anterograde mitochondria is much more than retrograde mitochondria in both areas (prox: antero: 12.7 ± 1.2% vs. retro: 2.7 ± 0.4%, *n* = 21 fish, *p* < 0.001; dist: antero: 17.1 ± 1.3% vs. retro: 5.7 ± 0.8%, *n* = 21 fish, Student’s two-tailed *t*-test, *p* < 0.001; **Figure 1E**). But the percentages of anterograde or retrograde mitochondria did not differ in these two areas (antero: prox: 81.8 ± 2.8% vs. dist: 74.1 ± 3.6%, *n* = 21 fish, Student’s two-tailed *t*-test, *p* = 0.104; **Figure 1F**). For moving speed, mitochondria in distal axons were faster than their counterparts in proximal axons regardless of the direction (antero: prox: 0.445 ± 0.010 μm/s, *n* = 230 mitos from 21 fish vs. dist: 0.483 ± 0.010 μm/s, *n* = 219 mitos from 21 fish, *p* = 0.007; retro: prox: 0.470 ± 0.028 μm/s, *n* = 44 mitos from 21 fish vs. dist: 0.555 ± 0.026 μm/s, *n* = 76 mitos from 21 fish, Student’s two-tailed *t*-test, *p* = 0.041; **Figure 1G**). Interestingly, retrogradely moving mitochondria were faster than those moving anterogradely in the distal axons (Student’s two-tailed *t*-test, *p* = 0.002; **Figure 1G**), while in proximal axons, there was no difference in speed (Student’s two-tailed *t*-test, *p* = 0.323, **Figure 1G**).

**FIGURE 2:**
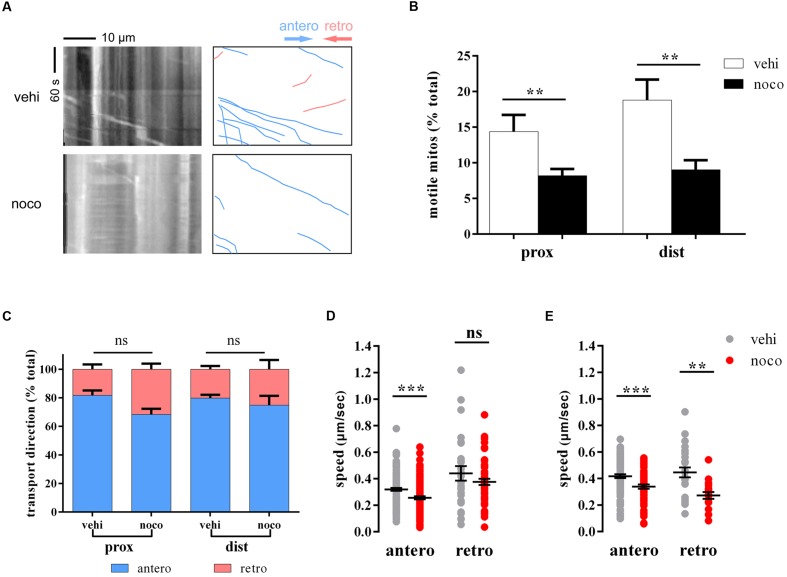
**Nocodazole treatment disrupts mitochondrial trafficking.** **(A)** The kymographs (left) depict moving mitochondria in vehicle- (top) and nocodazole-treated (below) zebrafish larvae at 6 dpf. Right kymographs are hand-drawn traces, and the diagonal lines represent moving mitochondria (anterograde, blue lines; retrograde, pink lines). **(B–E)** Mitochondrial motility **(B)**, transport direction (**C**; anterograde, blue; retrograde, pink), and speed (**D**, in the area of proximal axons; **E**, in the a rea of distal axons) in vehicle- (DMSO; gray) and nocodazole- (red) treated zebrafish larvae at 6 dpf. ^∗∗^*p* < 0.01, ^∗∗∗^*p* < 0.001. Error bars represent SEM.

Nocodazole can disrupt mitochondrial transport by interfering with microtubule polymerization (Plucinska et al., 2012). To verify whether that our model is suitable for analyzing mitochondrial transport in zebrafish M cells, we treated larvae with nocodazole at 6 dpf for 7 h. As expected, the percentage of moving mitochondria decreased significantly (prox: vehi: 14.4 ± 2.3%, *n* = 6 fish vs. noco: 8.2 ± 0.9%, *n* = 16 fish, Student’s two-tailed *t*-test, *p* = 0.007; dist: vehi: 18.8 ± 2.9%, *n* = 5 fish vs. noco: 9.0 ± 1.3%, *n* = 8 fish, Student’s two-tailed *t*-test, *p* = 0.005; **Figures 2A,B**). In addition, it was no evidence shown that the transport direction of mitochondria had obviously changed before and after nocodazole treatment (prox: antero: vehi: 81.7 ± 3.8%, *n* = 6 fish vs. noco: 68.4 ± 4.0%, *n* = 16 fish, Student’s two-tailed *t*-test, *p* = 0.0713; dist: antero: vehi: 79.8 ± 2.5%, *n* = 5 fish vs. noco: 74.9 ± 7.0%, *n* = 8 fish, Student’s two-tailed *t*-test, *p* = 0.607; **Figure 2C**). Mitochondrial speed in the nocodazole group was considerably slower in both transport directions and areas compared with the vehicle group (prox: antero: vehi: 0.319 ± 0.011 μm/s, *n* = 135 mitos from 6 fish vs. noco: 0.256 ± 0.012 μm/s, *n* = 131 mitos from 16 fish, Student’s two-tailed *t*-test, *p* < 0.001; dist: antero: vehi: 0.417 ± 0.014 μm/s, *n* = 97 mitos from 5 fish vs. noco: 0.338 ± 0.016 μm/s, *n* = 58 mitos from 8 fish, Student’s two-tailed *t*-test, *p* < 0.001; dist: retro: vehi: 0.446 ± 0.037 μm/s, *n* = 26 mitos from 5 fish vs. noco: 0.272 ± 0.026 μm/s, *n* = 18 mitos from 8 fish, Student’s two-tailed *t*-test, *p* = 0.001; **Figures 2D,E**), except retrogradely moving mitochondria in proximal axons did not reach significance (prox: retro: vehi: 0.440 ± 0.055 μm/s, *n* = 27 mitos from 6 fish vs. noco: 0.376 ± 0.023 μm/s, *n* = 55 mitos from 16 fish, Student’s two-tailed *t*-test, *p* = 0.204; **Figure 2D**). Therefore, labeled zebrafish could be used to quantitatively analyze axonal mitochondria dynamics in M axons under diverse physiological and pharmacological conditions via non-invasive *in vivo* imaging.

### Characterization of Mitochondrial Transport after Laser Axotomy

**FIGURE 3:**
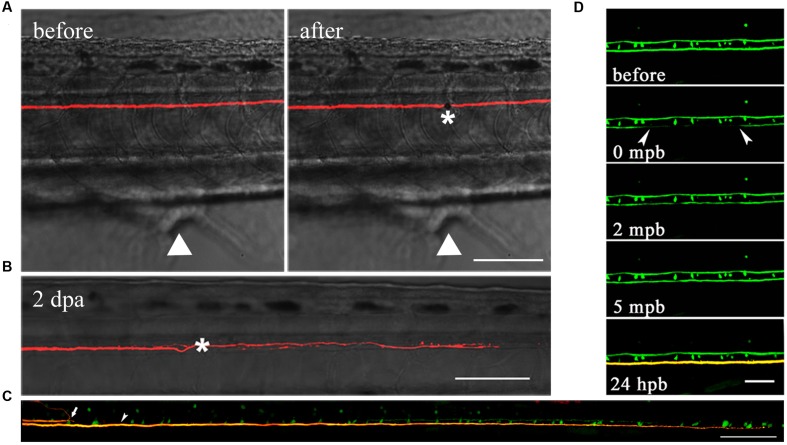
**M axons were completely severed by laser and have regenerative capacity after injury.** **(A)** M axons were axotomized by two-photon laser before (left) and after (right). Asterisk, injury site; white arrowheads, cloacal pores. **(B)** Injured axons regenerated to hundreds of microns 2 days after axotomy. Asterisk, injury site. **(C)** Using transgenic *Tg (Tol-056)* lines, the uncut axon (bottom) was longer than the severed axon (top) which stopped at the lesion site, implying that laser-induced injured axons were physically disconnected. White arrow, severed axon; white arrowhead, uncut axon. **(D)** One-photon laser was used to photobleach axons. Green fluorescence recovered after several minutes. When the photobleached axon was labeled with red dye at 24 hpb, its structure was still physically connected. Scale bars: **(A–C)** 100 μm, **(D)** 50 μm.

Laser axotomy has been widely used in zebrafish, especially in the PNS (O’Brien et al., 2009; O’Donnell et al., 2013; Vargas et al., 2015). Different from mechanical lesioning (Bhatt et al., 2004; Feng et al., 2010), we utilized a two-photon laser to transect M axons over cloacal pores at 6 dpf (**Figure 3A**). At 1–4 days after laser ablation, we found axons have relatively strong ability to extend through the injury site in comparison with mechanical transection by broken glass pipettes. The injured axon had regenerated to hundreds of microns 2 days after laser axotomy (**Figure 3B**). These results disagree with previous research suggesting that the regenerative capability of M axons is poor (Bhatt et al., 2004), and the biggest difference between each method is that we manipulate the lesion by laser rather than broken glass pipettes. In fact, laser axotomy can precisely cut a certain axon, while mechanical lesion almost severs large parts of the spinal cord. To demonstrate that axons were actually severed by laser rather than photobleached, we collected direct and indirect evidence (**Figures 3C,D**). First, an axon axotomized by two-photon laser should theoretically cause its physical disconnection. To examine this, we used transgenic *Tg (Tol-056)* lines that have a pair of M axons labeled by EGFP, and the unilateral axon was axotomized by laser. Several hours later, two M cells were electroporated with red dye (Alexa Fluor 594, Molecular Probes), so the axons should be yellow if they are connected. The uncut axon is longer than the severed axon, which stops at the lesion site, implying that laser-induced injured axons were physically disconnected (**Figure 3C**). Second, to demonstrate that laser-induced injured axons were not simply photobleached, one-photon laser was used to photobleach axons. Several minutes later, the green fluorescence recovered. In addition, when the one-photon photobleached axon was labeled with red dye at 24 h post-bleaching (hpb), the result illustrated that its structure was physically connected (**Figure 3D**). These results confirmed that M axons could be accurately severed by laser and that laser-induced injured axons were completely disconnected rather than photobleached.

**FIGURE 4:**
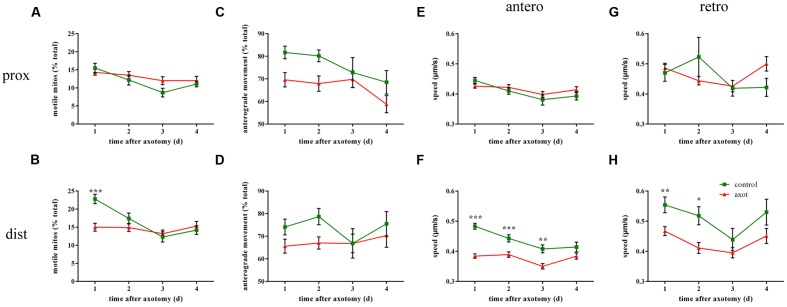
**Mitochondrial transport is affected following laser axotomy compared with uncut axons from age-matched controls.** **(A,C,E,G)** Line charts show mitochondrial motility **(A)**, the percentage of anterograde mitochondria **(C)**, and speed in each direction **(E**, anterograde; **G**, retrograde) in the area of proximal axons. **(B,D,F,H)** Transport parameters of mitochondria in distal axons are shown, including mitochondrial motility **(B)**, the percentage of anterograde mitochondria **(D)**, and speed in each direction (**F**, anterograde; **H**, retrograde). ^∗^*p* < 0.05, ^∗∗^*p* < 0.01, ^∗∗∗^*p* < 0.001. Error bars represent SEM.

To investigate whether mitochondrial transport was affected by laser axotomy, we observed axonal mitochondria in transected M axons during 4 days after injury and compared it with uncut axons from age-matched controls. In the area of proximal axons, injury did not affect mitochondrial trafficking including motility (two-way ANOVA, *p* ≥ 0.366, *n* ≥ 9 fish; **Figure 4A**), transport direction (two-way ANOVA, *p* ≥ 0.115, *n* ≥ 9 fish; **Figure 4C**), or speed (two-way ANOVA, antero: *p* ≥ 0.366, *n* ≥ 68 mitos; retro: *p* ≥ 0.192, *n* ≥ 28 mitos; **Figures 4E,G**). In contrast, mitochondrial transport closer to the injury site was markedly affected soon after axotomy. In distal axon areas, the percentage of moving mitochondria decreased dramatically at 1 dpa (two-way ANOVA, *p* < 0.001, *n* ≥ 21 fish; **Figure 4B**), but relative to the controls, overall mitochondrial motility recovered 2–4 dpa (two-way ANOVA, *p* ≥ 0.636, *n* ≥ 9 fish; **Figure 4B**). The percentage of mitochondria moving in the anterograde direction did not change every day after injury compared with the uncut group (two-way ANOVA, *p* ≥ 0.226, *n* ≥ 9 fish; **Figure 4D**). Regardless of direction, the speed has slowed in the early days in the laser-induced axotomy group (two-way ANOVA, antero: 1–3 dpa: *p* ≤ 0.006, *n* ≥ 83 mitos; 4 dpa: *p* = 0.353, *n* ≥ 81 mitos; retro: 1–2 dpa: *p* ≤ 0.013, *n* ≥ 41 mitos; 3–4 dpa: *p* ≥ 0.283, *n* ≥ 28 mitos; **Figures 4F,H**). Thus, laser axotomy in M cells could obviously interfere with mitochondrial transport in the distal area of axons, particularly in mitochondrial motility and speed in the early post-axotomy period.

### Correlations between Mitochondrial Transport Parameters and Axon Regenerative Capability

**FIGURE 5:**
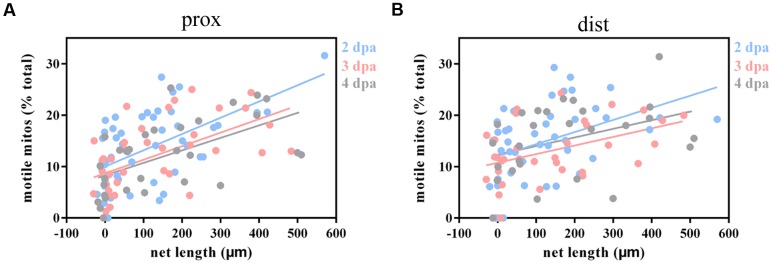
**Injured axons with stronger regenerative capacity are positively correlated with greater mitochondrial motility.** **(A,B)** Linear regression analysis between mitochondrial motility and the net length of corresponding axons at 2–4 dpa in proximal **(A)** and distal **(B)** axons. Blue, 2 dpa; pink, 3 dpa; gray, 4 dpa.

Considering that mitochondria play a vital role in complicated dynamic cellular processes and could be affected by axotomy, we were wondering whether mitochondrial dynamics correlate with axon regenerative capability, so we analyzed the relationship between moving mitochondria and the net length of correspondingly regenerative axons at 2–4 dpa (the calculation of net length at 1 dpa was inaccurate because of axon retraction). Linear regression analysis demonstrated a significant positive correlation between mitochondrial motility and axon regenerative capability (prox: 2 dpa: *R*^2^ = 0.3147, *p* < 0.001, *n* = 49 fish; 3 dpa: *R*^2^ = 0.3208, *p* < 0.001, *n* = 38 fish; 4 dpa: *R*^2^ = 0.3277, *p* < 0.001, *n* = 31 fish; dist: 2 dpa: *R*^2^ = 0.1783, *p* = 0.004, *n* = 44 fish; 3 dpa: *R*^2^ = 0.1962, *p* = 0.010, *n* = 33 fish; 4 dpa: *R*^2^ = 0.1439, *p* = 0.051, *n* = 27 fish; **Figures 5A,B**), except for the data in the distal axons at 4 dpa, which may be due to a small number of samples. These results indicate that the higher percentage of moving mitochondria is intimately related to stronger axon regenerative capability.

However, the regenerative capability of axons did not affect mitochondrial transport direction since linear regression analysis demonstrated that there was no relationship between regenerative capability and the percentage of anterograde mitochondria (prox: 2–4 dpa: *R*^2^ ≤ 0.0178, *p* ≥ 0.366, *n* ≥ 30 fish; dist: 2–4 dpa: *R*^2^ ≤ 0.0266, *p* ≥ 0.308, *n* ≥ 26 fish; **Supplementary Figures 1A,B**). Meanwhile, similar to the transport direction findings, mitochondrial speed in both directions did not significantly correlate with axon regenerative capability except for retrograde-moving mitochondria at 4 dpa (prox: antero: 2–4 dpa : *R*^2^ ≤ 0.0820, *p* ≥ 0.054, *n* ≥ 28 fish; retro: 2–4 dpa: *R*^2^ ≤ 0.0011, *p* ≥ 0.829, *n* ≥ 29 fish; dist: antero: 2–4 dpa: *R*^2^ ≤ 0.1054, *p* ≥ 0.090, *n* ≥ 25 fish; retro: 2–3 dpa: *R*^2^ ≤ 0.0695, *p* ≥ 0.193, *n* ≥ 26 fish; 4 dpa: *R*^2^ = 0.2468, *p* = 0.019, *n* = 22 fish; **Supplementary Figures 1C–F**).

Collectively, these results demonstrate that axons with stronger regenerative ability maintain higher mitochondrial motility. However, mitochondrial transport direction and speed were not clearly correlated with axon regenerative capability.

### db-cAMP Alters Mitochondrial Transport and Promotes Axon Regeneration

**FIGURE 6:**
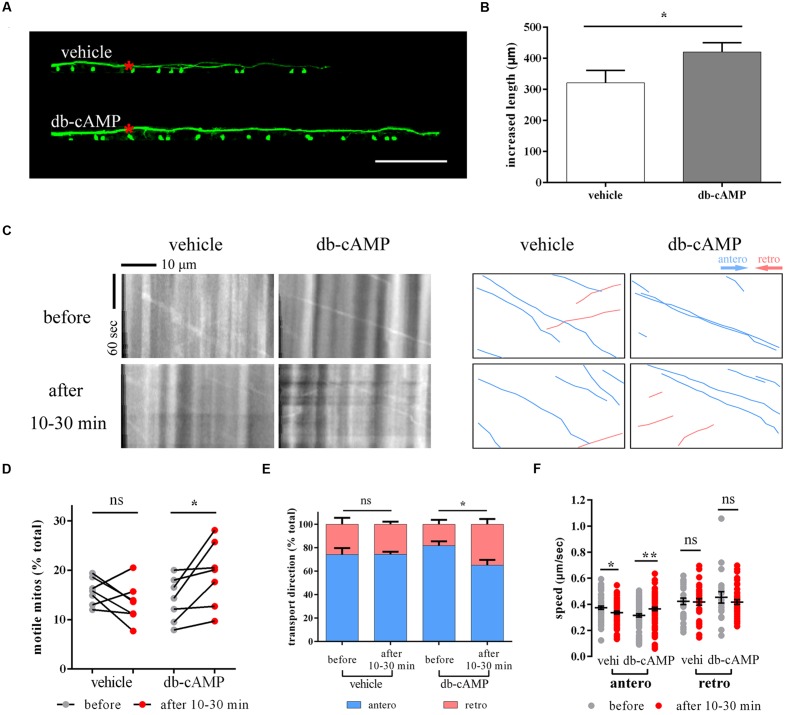
**Dibutyryl cyclic adenosine monophosphate boosts axon regeneration and promotes mitochondrial motility.** **(A,B)** The axon regeneration in vehicle- and db-cAMP-treated zebrafish at 2 dpa. **(C)** The kymographs display axonal transport of mitochondria before and after vehicle and db-cAMP treatments. Right kymographs are hand-drawn traces corresponding to the left kymographs (anterograde, blue lines; retrograde, pink lines). **(D–F)** Mitochondrial motility **(D)**, transport direction **(E)**, and speed **(F)** before and after vehicle and db-cAMP treatments. Scale bar: **(A)** 100 μm. ^∗^*p* < 0.05, ^∗∗^*p* < 0.01. Error bars represent SEM.

Dibutyryl cyclic adenosine monophosphate, a membrane-permeable analog of endogenous cAMP, can promote axon regeneration *in vitro* and *in vivo* (Qiu et al., 2002; Bhatt et al., 2004; Monsul et al., 2004). It has been reported that db-cAMP can promote M axon regeneration and restore function in living zebrafish (Bhatt et al., 2004). Using *Tg (Tol-056)* lines (Satou et al., 2009), we confirmed that db-cAMP facilitates axon regeneration 2 days after laser ablation (vehi: 321.4 ± 39.7 μm, *n* = 31 fish vs. db-cAMP: 420.6 ± 30.0 μm, *n* = 36 fish, Student’s one-tailed *t*-test, *p* = 0.024; **Figures 6A,B**). Next, to determine whether db-cAMP could also impact mitochondrial transport, we microinjected db-cAMP or vehicle into the hindbrain of electroporated zebrafish at 8 dpf and imaged mitochondria in distal axons to monitor mitochondrial transport before and after treatment. Based on previous results (**Figures 1E–G** and **2B–E**), the tendency for changes in mitochondrial trafficking in proximal and distal axons is similar in non-axotomized M cells under different physiological and pharmacological conditions, so only selecting distal axons is a reliable way to monitor changes in mitochondrial transport.

Between 10 and 30 min after microinjection, mitochondrial motility appeared to rise in db-cAMP-treated zebrafish larvae (before: 14.0 ± 1.7% vs. after: 19.2 ± 2.5%, *n* = 7 fish, paired Student’s two-tailed *t*-test, *p* = 0.035; **Figures 6C,D**), but not in the vehicle-treated group (before: 15.7 ± 1.0% vs. after: 13.4 ± 1.5%, *n* = 7 fish, paired Student’s two-tailed *t*-test, *p* = 0.213; **Figures 6C,D**). The moving direction of mitochondria in the db-cAMP-treated group also changed, with mitochondrial trafficking at a lower proportion of the anterogradely moving between 10 and 30 min after microinjection (antero: before: 81.7 ± 4.0%, *n* = 7 fish vs. after: 65.1 ± 4.9%, *n* = 7 fish, Student’s two-tailed *t*-test, *p* = 0.022; **Figure 6E**), while there was no difference before and after vehicle solution treatment (antero: before: 74.1 ± 6.0%, *n* = 7 fish vs. after: 74.3 ± 2.4%, *n* = 7 fish, Student’s two-tailed *t*-test, *p* = 0.978; **Figure 6E**). The speed of anterograde mitochondria was faster in the db-cAMP-treated group 10–30 min after microinjection (before: 0.315 ± 0.013 μm/s, *n* = 69 mitos from 7 fish vs. after: 0.364 ± 0.012 μm/s, *n* = 85 mitos from 7 fish, Student’s two-tailed *t*-test, *p* = 0.007; **Figure 6F**), but the retrograde speed was unchanged (before: 0.453 ± 0.044 μm/s, *n* = 20 mitos from 7 fish vs. after: 0.417 ± 0.018 μm/s, *n* = 43 mitos from 7 fish, Student’s two-tailed *t*-test, *p* = 0.369; **Figure 6F**). Consequently, these results indicate that db-cAMP accelerated axon growth after injury and also affected mitochondrial transport, particularly in mitochondrial motility.

## Discussion

We developed a novel model to clarify the relationship between axon regenerative capacity and inner mitochondrial transport in the vertebrate CNS using non-invasive *in vivo* imaging at the single-axon level. Our results are the first evidence that laser-induced injured M axons can robustly regenerate. By integrating single-cell electroporation with *in vivo* laser axotomy, we demonstrated that the stronger regenerative capability of axons appears to be positively correlated with greater mitochondrial motility. Next, we used db-cAMP to stimulate the regenerative capacity of injured axons. Our data suggest that after db-cAMP treatment, the injured M axons displayed vigorous regeneration, accompanied with increased mitochondrial motility. Consequently, our model offers new insight into the connection between axon regeneration and mitochondria and paves the way to deeply understand a potential therapeutic avenue of axon regeneration by enhancing mitochondrial motility.

Mitochondria participate in many processes involving in neural survival and function (Sheng, 2014). There are many models for studying mitochondrial transport in living animal axons, including *Drosophila* (Pilling et al., 2006) mammals (Misgeld et al., 2007; Sorbara et al., 2014; Takihara et al., 2015; Zhou et al., 2016), and others. Because of their optical transparency and small size, researchers have come to realize that zebrafish are useful organisms for exploring mitochondria from transport to function without intricate surgery before direct microscope imaging (Plucinska et al., 2012; O’Donnell et al., 2013; Vargas et al., 2015; Bergamin et al., 2016; Dukes et al., 2016). However, none of those models allowed the observation of mitochondrial transport in the vertebrate spinal cord at a single-axon level through non-invasive *in vivo* time-lapse imaging until now, making it impossible to explore the correlation between axon regeneration and internal mitochondrial transport in the living vertebrate CNS. Here, we successfully labeled M cells and their inner mitochondria after hatching. M axons nearly parallel the zebrafish anterior–posterior axis through the spinal cord (Bhatt et al., 2004; Feng et al., 2010), which facilitates maintaining the focal *x*–*y* plane, and a single labeled axon is convenient for tracking axonal morphologic changes of regeneration and mitochondrial dynamics over a few days in specific axon areas. To ensure that labeled zebrafish larvae are fit for assaying changes in mitochondrial transport, nocodazole was used to restrict mitochondrial movement (Plucinska et al., 2012). As expected, nocodazole effectively constrained mitochondrial transport in M axons. Hence, our model provides a novel living vertebrate model to directly monitor and quantify mitochondria dynamics via a non-invasive approach in the CNS at a single-cell level under physiological or pharmacological conditions.

We initially applied *in vivo* laser axotomy (Yanik et al., 2004; O’Brien et al., 2009; Byrne et al., 2011; O’Donnell et al., 2013) to precisely sever a single M axon. Surprisingly, this methodology gave new evidence that injured M axons appear to regenerate more strongly than previous reports that M axons were transected with broken glass pipettes (Bhatt et al., 2004; Feng et al., 2010). We confirmed that M axons were completely severed by two-photon laser rather than photobleached. Previous publications pointed out that two types of elements are involved in axon regeneration (Benarroch, 2015): extrinsic factors outside cells including inhibitory molecules and intrinsic factors inside cells that can suppress axon regrowth (Bhatt et al., 2004). Discrepant regenerative capability of injured M axons might result from the different types of axotomy that cause distinctive and complicated CNS environments with various extrinsic and intrinsic factors. We suggest that extrinsic factors including the degree of inflammation may be influential following mechanical transection, which damages the surrounding tissue, but this needs further study. Therefore, we could conclude that laser-induced axotomy is more precise and less damaging than conventional mechanical transection. Most importantly, the laser-induced injured M axons exhibit stronger regenerative capacity.

Laser axotomy affected mitochondrial transport including motility and speed in the areas of distal axons near the injured site. These results are consistent with previous work showing that axonal injury disrupts mitochondrial transport *in vitro*, presumably by increasing the rate of stationary mitochondria and decreasing the speed of mitochondrial transport (Yokota et al., 2015). Considering the importance of mitochondria, we hypothesized that axon regenerative capability is correlated with mitochondrial transport. To test this, linear regression analysis was used to evaluate correlations between axon regenerative ability and mitochondrial transport parameters. Encouragingly, the results showed that mitochondrial trafficking was increased in injured axons with stronger regenerative capability. Similarly, published work indicated that mitochondrial fluxes mainly kept in high levels after injury in mice transected intercostal nerves that mounted a vigorous regrowth response (Misgeld et al., 2007). The relationship between regenerative capability and mitochondrial speed or transport direction were not analyzed in this model. We speculate that axons with stronger regenerative ability require more energy than those weaker regenerative ability (Zhou et al., 2016). As a result of increased energy needs, more mitochondria are moved to provide sources to produce ATP. Mitochondrial speed and transport direction, however, are not directly related to supplying energy. Therefore, our results imply that robust axon regeneration with more motivated mitochondrial motility is conserved among vertebrates, and laser-induced axotomy of M cells with labeled mitochondria is a useful model for studying the interaction between axon regeneration and mitochondria at a single-axon level through non-invasive *in vivo* imaging. Many avenues of study could be explored using this model. For example, mitochondrial activity, a significant index of mitochondrial function, could be detected using biosensors such as mitochondrial relative changes in pH values (Chen et al., 2015), redox potential (Hanson et al., 2004; O’Donnell et al., 2013), and calcium concentration (Pozzan and Rudolf, 2009). Such investigations will clarify the connections between axon regeneration and mitochondria from their trafficking to functions.

Additionally, we wondered whether enhancing axon regeneration would improve mitochondrial motility. To explore this hypothesis, we used db-cAMP to regulate axon regeneration. cAMP has numerous targets that regulate cellular signaling (Qiu et al., 2002; Pearse et al., 2004; Valsecchi et al., 2013). As an analog to cAMP, db-cAMP has been reported to promote M axon regeneration in living zebrafish (Bhatt et al., 2004) and activate protein kinase A signaling in the rodent dorsal root ganglia, which could increase mitochondrial transport (Hiruma et al., 2002; Gibbs et al., 2015). Similarly, our research demonstrated that db-cAMP could improve injured axon regeneration and increase mitochondrial motility in living zebrafish larvae. Mitochondrial transport direction and speed were also affected by db-cAMP. This does not mean there was a contradiction between rising mitochondrial motility and decreased percentage of anterograde mitochondria; as mentioned above, transport direction here indicates the number of anterograde mitochondria divided by the total number of moving mitochondria. When individually analyzing the number of anterograde or retrograde mitochondria divided by the total number of mitochondria (number of anterograde or retrograde mitochondria/total number of mitochondria, A/T or R/T), we found that both the number of anterograde and retrograde mitochondria relatively increased after db-cAMP treatment (before: A/T = 11.1%; R/T = 2.9%, *n* = 7 fish vs. after: A/T = 12.8%; R/T = 6.4%, *n* = 7 fish), which is in accordance with the finding that db-cAMP could raise mitochondrial motility. However, the tendencies of changes in anterograde and retrograde mitochondrial speed after db-cAMP treatment varied, and the mechanism is not fully understood. On the other hand, new research (Zhou et al., 2016) showed that genetic manipulation to enhance mitochondrial movement could facilitate axon regeneration *in vivo* and *in vitro*. Thus, we look forward to further investigations into the regulation of mitochondrial motility or axon regrowth via gene expression or repression genes in this model, which may elucidate the interactions between axon regeneration and mitochondria *in vivo*.

In summary, this unique and promising model provides valuable data that clarify relationship between axon regeneration and mitochondrial transport in living zebrafish CNS from a single-cell perspective.

## Author Contributions

Conceived and designed the experiments: YX, MC, BbH, and BH. Performed the experiments: YX, MC, BbH, and RH. Analyzed the data: YX and MC. Contributed reagents/materials/analysis tools: YX, MC, BbH, RH, and BH. Wrote the paper: YX and BH.

## Conflict of Interest Statement

The authors declare that the research was conducted in the absence of any commercial or financial relationships that could be construed as a potential conflict of interest.

## References

[B1] Benarroch E. E. (2015). Acquired axonal degeneration and regeneration: recent insights and clinical correlations. *Neurology* 84 2076–2085. 10.1212/WNL.0000000000001601 25904690

[B2] Bergamin G. Cieri D. Vazza G. Argenton F. Mostacciuolo M. L. (2016). Zebrafish Tg(hb9:MTS-Kaede): a new in vivo tool for studying the axonal movement of mitochondria. *Biochim. Biophys. Acta* 1860 1247–1255. 10.1016/j.bbagen.2016.03.007 26968460

[B3] Bestman J. E. Ewald R. C. Chiu S. L. Cline H. T. (2006). In vivo single-cell electroporation for transfer of DNA and macromolecules. *Nat. Protoc.* 1 1267–1272. 10.1038/nprot.2006.186 17406410

[B4] Bhatt D. H. Otto S. J. Depoister B. Fetcho J. R. (2004). Cyclic AMP-induced repair of zebrafish spinal circuits. *Science* 305 254–258. 10.1126/science.1098439 15247482

[B5] Bolea I. Gan W. B. Manfedi G. Magrane J. (2014). Imaging of mitochondrial dynamics in motor and sensory axons of living mice. *Methods Enzymol.* 547 97–110. 10.1016/B978-0-12-801415-8.00006-0 25416354

[B6] Byrne A. B. Edwards T. J. Hammarlund M. (2011). In vivo laser axotomy in *C. elegans*. *J. Vis. Exp.* 51:2707 10.3791/2707 PMC3168200 21633331

[B7] Canty A. J. Huang L. Jackson J. S. Little G. E. Knott G. Maco B. (2013). In-vivo single neuron axotomy triggers axon regeneration to restore synaptic density in specific cortical circuits. *Nat. Commun.* 4:2038 10.1038/ncomms3038 23799397

[B8] Chen M. Zheng B. (2014). Axon plasticity in the mammalian central nervous system after injury. *Trends Neurosci.* 37 583–593. 10.1016/j.tins.2014.08.008 25218468 PMC4190162

[B9] Chen Y. Zhu C. Cen J. Bai Y. He W. Guo Z. (2015). Ratiometric detection of pH fluctuation in mitochondria with a new fluorescein/cyanine hybrid sensor. *Chem. Sci.* 6 3187–3194. 10.1039/C4SC04021J PMC5490428 28706690

[B10] Court F. A. Coleman M. P. (2012). Mitochondria as a central sensor for axonal degenerative stimuli. *Trends Neurosci.* 35 364–372. 10.1016/j.tins.2012.04.001 22578891

[B11] Distel M. Wullimann M. F. Koster R. W. (2009). Optimized Gal4 genetics for permanent gene expression mapping in zebrafish. *Proc. Natl. Acad. Sci. U.S.A.* 106 13365–13370. 10.1073/pnas.0903060106 19628697 PMC2726396

[B12] Dukes A. A. Bai Q. Van Laar V. S. Zhou Y. Ilin V. David C. N. (2016). Live imaging of mitochondrial dynamics in CNS dopaminergic neurons in vivo demonstrates early reversal of mitochondrial transport following MPP(+) exposure. *Neurobiol. Dis.* 95 238–249. 10.1016/j.nbd.2016.07.020 27452482 PMC5010936

[B13] Feng Y. Yan T. Zheng J. Ge X. Mu Y. Zhang Y. (2010). Overexpression of Wld(S) or Nmnat2 in mauthner cells by single-cell electroporation delays axon degeneration in live zebrafish. *J. Neurosci. Res.* 88 3319–3327. 10.1002/jnr.22498 20857515

[B14] Gibbs K. L. Greensmith L. Schiavo G. (2015). Regulation of Axonal Transport by Protein Kinases. *Trends Biochem. Sci.* 40 597–610. 10.1016/j.tibs.2015.08.003 26410600

[B15] Haas K. Sin W. C. Javaherian A. Li Z. Cline H. T. (2001). Single-cell electroporation for gene transfer in vivo. *Neuron* 29 583–591. 10.1016/S0896-6273(01)00235-5 11301019

[B16] Hanson G. T. Aggeler R. Oglesbee D. Cannon M. Capaldi R. A. Tsien R. Y. (2004). Investigating mitochondrial redox potential with redox-sensitive green fluorescent protein indicators. *J. Biol. Chem.* 279 13044–13053. 10.1074/jbc.M312846200 14722062

[B17] Hiruma H. Saito A. Kusakabe T. Takenaka T. Kawakami T. (2002). Neuropeptide Y inhibits axonal transport of particles in neurites of cultured adult mouse dorsal root ganglion cells. *J. Physiol.* 543 85–97. 10.1113/jphysiol.2002.020578 12181283 PMC2290469

[B18] Huang Y. B. Hu C. R. Zhang L. Yin W. Hu B. (2015). In vivo study of dynamics and stability of dendritic spines on olfactory bulb interneurons in *Xenopus laevis* tadpoles. *PLoS ONE* 10:e0140752 10.1371/journal.pone.0140752 PMC4617280 26485435

[B19] Kerschensteiner M. Schwab M. E. Lichtman J. W. Misgeld T. (2005). In vivo imaging of axonal degeneration and regeneration in the injured spinal cord. *Nat. Med.* 11 572–577. 10.1038/nm1229 15821747

[B20] Kim M. J. Kang K. H. Kim C. H. Choi S. Y. (2008). Real-time imaging of mitochondria in transgenic zebrafish expressing mitochondrially targeted GFP. *Biotechniques* 45 331–334. 10.2144/000112909 18778258

[B21] Korn H. Faber D. S. (2005). The Mauthner cell half a century later: a neurobiological model for decision-making? *Neuron* 47 13–28. 10.1016/j.neuron.2005.05.019 15996545

[B22] Lathrop K. L. Steketee M. B. (2013). Mitochondrial dynamics in retinal ganglion cell axon regeneration and growth cone guidance. *J. Ocul. Biol.* 1:9. PMC3946936 24616897

[B23] Li Z. Okamoto K. Hayashi Y. Sheng M. (2004). The importance of dendritic mitochondria in the morphogenesis and plasticity of spines and synapses. *Cell* 119 873–887. 10.1016/j.cell.2004.11.003 15607982

[B24] Lorenzana A. O. Lee J. K. Mui M. Chang A. Zheng B. (2015). A surviving intact branch stabilizes remaining axon architecture after injury as revealed by in vivo imaging in the mouse spinal cord. *Neuron* 86 947–954. 10.1016/j.neuron.2015.03.061 25937174 PMC4441580

[B25] Miller K. E. Sheetz M. P. (2004). Axonal mitochondrial transport and potential are correlated. *J. Cell Sci.* 117 2791–2804. 10.1242/jcs.01130 15150321

[B26] Misgeld T. Kerschensteiner M. Bareyre F. M. Burgess R. W. Lichtman J. W. (2007). Imaging axonal transport of mitochondria in vivo. *Nat. Methods* 4 559–561. 10.1038/nmeth1055 17558414

[B27] Monsul N. T. Geisendorfer A. R. Han P. J. Banik R. Pease M. E. Skolasky R. L. (2004). Intraocular injection of dibutyryl cyclic AMP promotes axon regeneration in rat optic nerve. *Exp. Neurol.* 186 124–133. 10.1016/S0014-4886(03)00311-X 15026251

[B28] O’Brien G. S. Rieger S. Martin S. M. Cavanaugh A. M. Portera-Cailliau C. Sagasti A. (2009). Two-photon axotomy and time-lapse confocal imaging in live zebrafish embryos. *J. Vis. Exp.* 24:1129 10.3791/1129 PMC2726581 19229185

[B29] O’Donnell K. C. Vargas M. E. Sagasti A. (2013). WldS and PGC-1alpha regulate mitochondrial transport and oxidation state after axonal injury. *J. Neurosci.* 33 14778–14790. 10.1523/JNEUROSCI.1331-13.2013 24027278 PMC3771034

[B30] Paquet D. Plucinska G. Misgeld T. (2014). In vivo imaging of mitochondria in intact zebrafish larvae. *Methods Enzymol.* 547 151–164. 10.1016/B978-0-12-801415-8.00009-6 25416357

[B31] Pearse D. D. Pereira F. C. Marcillo A. E. Bates M. L. Berrocal Y. A. Filbin M. T. (2004). cAMP and Schwann cells promote axonal growth and functional recovery after spinal cord injury. *Nat. Med.* 10 610–616. 10.1038/nm1056 15156204

[B32] Pilling A. D. Horiuchi D. Lively C. M. Saxton W. M. (2006). Kinesin-1 and Dynein are the primary motors for fast transport of mitochondria in *Drosophila* motor axons. *Mol. Biol. Cell* 17 2057–2068. 10.1091/mbc.E05-06-0526 16467387 PMC1415296

[B33] Plucinska G. Paquet D. Hruscha A. Godinho L. Haass C. Schmid B. (2012). In vivo imaging of disease-related mitochondrial dynamics in a vertebrate model system. *J. Neurosci.* 32 16203–16212. 10.1523/JNEUROSCI.1327-12.2012 23152604 PMC6794024

[B34] Pozzan T. Rudolf R. (2009). Measurements of mitochondrial calcium in vivo. *Biochim. Biophys. Acta* 1787 1317–1323. 10.1016/j.bbabio.2008.11.012 19100709

[B35] Qiu J. Cai D. Dai H. McAtee M. Hoffman P. N. Bregman B. S. (2002). Spinal axon regeneration induced by elevation of cyclic AMP. *Neuron* 34 895–903. 10.1016/S0896-6273(02)00730-4 12086638

[B36] Russo G. J. Louie K. Wellington A. Macleod G. T. Hu F. Panchumarthi S. (2009). *Drosophila* Miro is required for both anterograde and retrograde axonal mitochondrial transport. *J. Neurosci.* 29 5443–5455. 10.1523/JNEUROSCI.5417-08.2009 19403812 PMC2693725

[B37] Sajic M. Mastrolia V. Lee C. Y. Trigo D. Sadeghian M. Mosley A. J. (2013). Impulse conduction increases mitochondrial transport in adult mammalian peripheral nerves in vivo. *PLoS Biol.* 11:e1001754 10.1371/journal.pbio.1001754 PMC3876979 24391474

[B38] Satou C. Kimura Y. Kohashi T. Horikawa K. Takeda H. Oda Y. (2009). Functional role of a specialized class of spinal commissural inhibitory neurons during fast escapes in zebrafish. *J. Neurosci.* 29 6780–6793. 10.1523/JNEUROSCI.0801-09.2009 19474306 PMC6665578

[B39] Saxton W. M. Hollenbeck P. J. (2012). The axonal transport of mitochondria. *J. Cell Sci.* 125 2095–2104. 10.1242/jcs.053850 22619228 PMC3656622

[B40] Schwarz T. L. (2013). Mitochondrial trafficking in neurons. *Cold Spring Harb. Perspect. Biol.* 5:a011304 10.1101/cshperspect.a011304 PMC3660831 23732472

[B41] Sheng Z. H. (2014). Mitochondrial trafficking and anchoring in neurons: new insight and implications. *J. Cell Biol.* 204 1087–1098. 10.1083/jcb.201312123 24687278 PMC3971748

[B42] Sorbara C. D. Wagner N. E. Ladwig A. Nikic I. Merkler D. Kleele T. (2014). Pervasive axonal transport deficits in multiple sclerosis models. *Neuron* 84 1183–1190. 10.1016/j.neuron.2014.11.006 25433639

[B43] Steketee M. B. Moysidis S. N. Weinstein J. E. Kreymerman A. Silva J. P. Iqbal S. (2012). Mitochondrial dynamics regulate growth cone motility, guidance, and neurite growth rate in perinatal retinal ganglion cells in vitro. *Invest. Ophthalmol. Vis. Sci.* 53 7402–7411. 10.1167/iovs.12-10298 23049086 PMC3484733

[B44] Takihara Y. Inatani M. Eto K. Inoue T. Kreymerman A. Miyake S. (2015). In vivo imaging of axonal transport of mitochondria in the diseased and aged mammalian CNS. *Proc. Natl. Acad. Sci. U.S.A.* 112 10515–10520. 10.1073/pnas.1509879112 26240337 PMC4547257

[B45] Valsecchi F. Ramos-Espiritu L. S. Buck J. Levin L. R. Manfredi G. (2013). cAMP and mitochondria. *Physiology (Bethesda)* 28 199–209. 10.1152/physiol.00004.2013 23636265 PMC3870303

[B46] Vargas M. E. Yamagishi Y. Tessier-Lavigne M. Sagasti A. (2015). Live imaging of calcium dynamics during axon degeneration reveals two functionally distinct phases of calcium influx. *J. Neurosci.* 35 15026–15038. 10.1523/JNEUROSCI.2484-15.2015 26558774 PMC4642236

[B47] Yanik M. F. Cinar H. Cinar H. N. Chisholm A. D. Jin Y. Ben-Yakar A. (2004). Neurosurgery: functional regeneration after laser axotomy. *Nature* 432:822 10.1038/432822a 15602545

[B48] Yokota S. Takihara Y. Arimura S. Miyake S. Takamura Y. Yoshimura N. (2015). Altered transport velocity of axonal mitochondria in retinal ganglion cells after laser-induced axonal injury in vitro. *Invest. Ophthalmol. Vis. Sci.* 56 8019–8025. 10.1167/iovs.15-17876 26720449

[B49] Zhou B. Yu P. Lin M. Y. Sun T. Chen Y. Sheng Z. H. (2016). Facilitation of axon regeneration by enhancing mitochondrial transport and rescuing energy deficits. *J. Cell Biol.* 214 103–119. 10.1083/jcb.201605101 27268498 PMC4932375

