# Supplementary material for: In vivo Imaging of Mitochondrial Transport in Single-Axon Regeneration of Zebrafish Mauthner Cells
Source: Front Cell Neurosci. 2017 Jan 24;11:4. doi: 10.3389/fncel.2017.00004 (PMC5258718; doi:10.3389/fncel.2017.00004)
Supplement: Supplementary file 1 [file Presentation_1.PDF]

In vivo imaging of mitochondrial transport in single-axon regeneration of zebrafish Mauthner cells

Yang Xu[1†], Min Chen[1†], Bingbing Hu[1†], Rongchen Huang[1], Bing Hu[1*]

Chinese Academy of Sciences Key Laboratory of Brain Function and Disease, and School of Life Sciences, University of Science and Technology of China, Hefei, Anhui Province, P. R. China

[†] These authors have contributed equally to this work.

*** Correspondence:**

Bing Hu

bhu@ustc.edu.cn

Supplementary figure captions

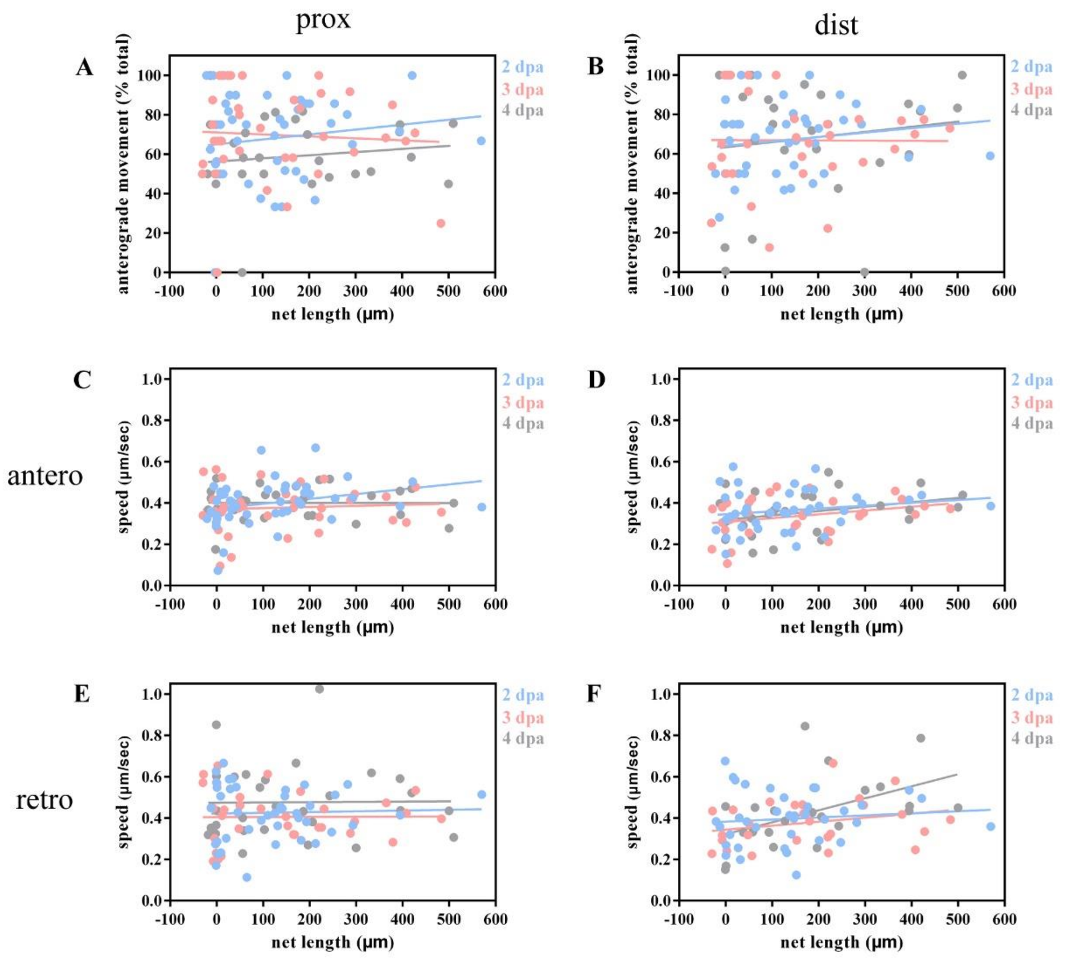

SUPPLEMENTARY FIGURE 1 Transport direction and speed of mitochondria are not correlated with the regenerative capability of individual axons. **(A, B)** Linear regression analysis of the percentage of anterograde mitochondria according to the net

length of corresponding axons at 2-4 dpa in individual proximal **(A)** and distal **(B)** axons. **(C-F)** Linear regression analysis between mitochondrial speed and the net length of corresponding axons at 2-4 dpa. **(C)** Anterogradely moving mitochondria in proximal axons. **(D)** Anterogradely moving mitochondria in distal axons. **(E)** Retrogradely moving mitochondria in proximal axons. **(F)** Retrogradely moving mitochondria in distal axons. Blue, 2 dpa; pink, 3 dpa; grey, 4 dpa.
